# Properties of Orthopaedic Cements Biomechanically Little Affected by Exceptional Use of Liquid Antibiotics

**DOI:** 10.1111/os.12911

**Published:** 2021-10-04

**Authors:** Hetzmannseder Sophie, Chang Yuhan, Kittinger Clemens, Kühn Klaus‐Dieter

**Affiliations:** ^1^ Department of Orthopaedics and Trauma Medical University of Graz Graz Austria; ^2^ Department of Orthopaedic Surgery Chang Gung Memorial Hospital Taoyuan Taiwan; ^3^ Institute of Hygiene Microbiology and Environmental Medicine, Medical University of Graz Graz Austria

**Keywords:** Antibiotics, Biomechanics, Clindamycin, Efficacy, PMMA bone cement

## Abstract

**Objectives:**

To specify the concentration of the liquid antibiotics to be added to polymethylmethacrylate (PMMA) and its impact on the quality of the spacer is the purpose of this study with liquid clindamycin added to different cements.

**Methods:**

In the present study, eight different cement mixtures were prepared and investigated. In the following, number 1 indicates the references, 2 all cements after liquid clindamycin was added to the liquid cement compound, 3 all cements after liquid clindamycin was added to the cement powder, and 4 all cements after liquid clindamycin was added to the cement dough. After curing, cements were filled into metal moulds and a pressure of 3 bar was maintained for 30 min. Mechanical investigations were carried out according to ISO 5833 (2002) and DIN 53435 (2007). For microbiological tests, standardized cylindrical mouldings (diameter: 25 mm, height: 10 mm) were produced and incubated in 10 ml buffer solution at room temperature for 24 h. All eluates were generated by spreading previously established suspensions of *Staphylococcus aureus*, *Staphylococcus epidermidis*, *Cutibacterium acnes* and methicillin‐resistant *Staphylococcus aureus* (MRSA) with a 0.5 McFarland turbidity standard.

**Results:**

Apparently, we found that in all investigated cases, the admixture of liquid antibiotic negatively affected the mechanical characteristics of the cement mould. Among the various test groups, the influence on the ISO compression strength and ISO flexural modulus of the investigated test groups was only minimal when liquid clindamycin was added to cement liquid. Compared to admixing of liquid clindamycin into cement powder or dough ISO compression strength and ISO flexural modulus and flexural strength showed the maximum reduction. The efficacy against chosen germs was reduced as well when liquid antibiotic was admixed instead of powder. This admixture of liquid anti‐infective agents resulted in a 234% enhanced elution after 10 days 29 a negative effect on the inhibition zones were detected during the previous period.

**Conclusion:**

The admixture of powdery antibiotic is preferable to liquid antibiotics. If no powdery antibiotic is available, we can recommend the admixture of liquid antibiotic to liquid cement prior to dough production in case powdery antibiotics cannot be used. However, we discourage the admixture of liquid antibiotic to cement powder or cement dough during early low viscose phase.

## Graphical abstract

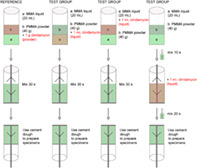

## Introduction

Endoprosthetic revision surgeries follow specified algorithms^1^, ^2^, ^3^. In addition to surgery as a therapeutic measure, systemic and local antibiotics (AB) are given as an accompanying adjuvant therapy^4^, ^5^, ^7^. Polymethylmethacrylate Bone cements based on polymethylmethacrylate (PMMA) spacers are often used as temporary placeholders in two‐stage surgeries^8^, ^9^. Depending on anamnesis and diagnosis, AB are manually added to the PMMA cement during the manufacturing process to ensure a high local drug concentration^10^, ^11^, ^12^.

The manual addition of AB is generally necessary if no customary PMMA cements with the required antibiotics for a particular surgery are commercially available^12^, ^13^, ^14^.

Anti‐infective agents are often applied perioperatively during the cement preparation^1^. The agent powder is added fractionally to the PMMA powder^13^, ^15^. Subsequently, the homogenized agent/PMMA powder is mixed with the liquid component, forming the cement dough^16^, ^17^. Adding of the antibiotic powder is highly effective because of their optimal release^11^, ^17^, ^18^, ^19^, ^20^. Admixing of powdery, sterile AB is based on recommendations taking mechanical stability, homogeneity, and reproducibility of drug release (e.g. PRO‐IMPLAN Foundation recommendation) into account^21^, ^22^. The recommended upper limit for the addition of AB is 4 g AB per 40 g cement powder (10% rule^23^).

Adding of liquid AB is commonly discouraged because it has a considerable impact on the mechanical stability^7^, ^15^, ^24^, ^25^, ^26^. However, Chang *et al*. (2011)^26^ describe good release rates admixing high concentrations (2 g of agent in 12 mL distilled water) of liquid gentamicin and vancomycin to PMMA, with moderate reduction of mechanic stability.

If a suitable sterile AB powder is not available, use of a liquid AB is necessary. Furthermore, if anti‐infective agents (e.g. antimycotics) are characterized by a poor elution for physical (hydrophobic) or chemical (cross‐links with PMMA) reasons, a liquid agent may be a possible alternative. Antifungal drugs have not received much attention when it comes to admixing.^27^ Chang *et al*. (2014)^28^ added liquid amphotericin B (2 g/12 mL with xylitol) to the liquid monomer and found sufficient release with improved efficacy against *Candida albicans*. Currently, there are no specifications concerning concentration and dose of the liquid AB to be added, let alone its impact on the quality of the spacer *in vivo*.

In this study, liquid clindamycin was added in low concentrations (1 g powder in 1 mL sterile water) to the cement liquid, the cement powder, and the cement dough during the early low viscose phase. The cements used were PALACOS R and PALACOS R + G. As reference, powdery clindamycin (1 g) was admixed. All manually manufactured cements were investigated mechanically according to International Standard Operations (ISO) 5833 (2002)^29^ or Deutsche Industrie Norm (DIN) 53435 (2007), as well as microbiologically with the inhibition zone test.

## Methods

### Manually Added Cement Mixtures

**Figure 1:**
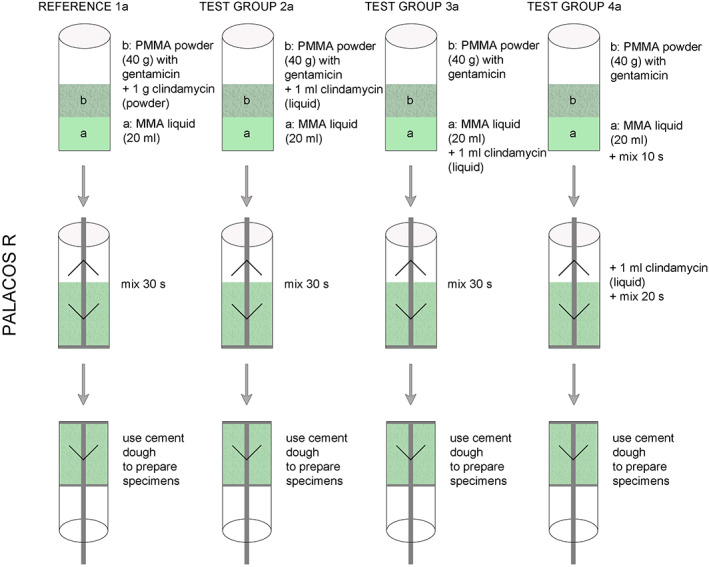
Experimental arrangement with Palacos R leading to the results (Fig. 3).

**Figure 2:**
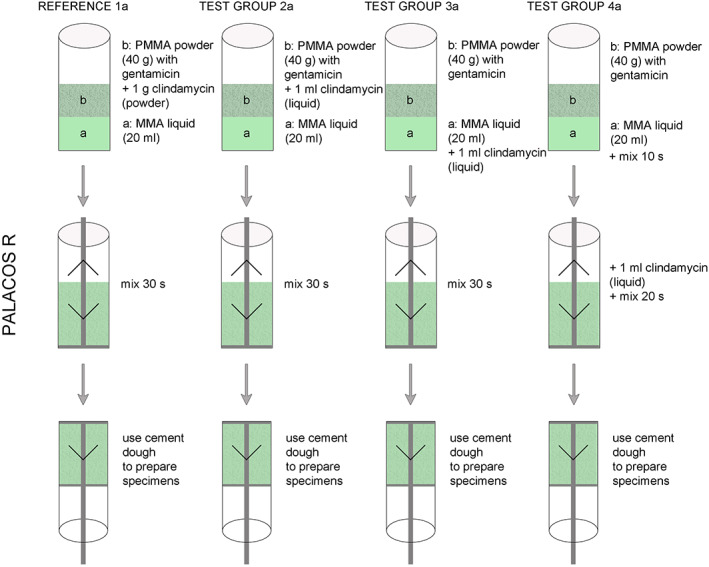
Experimental arrangement with Palacos R + G leading to the results (Fig. 3).

**Figure 3:**
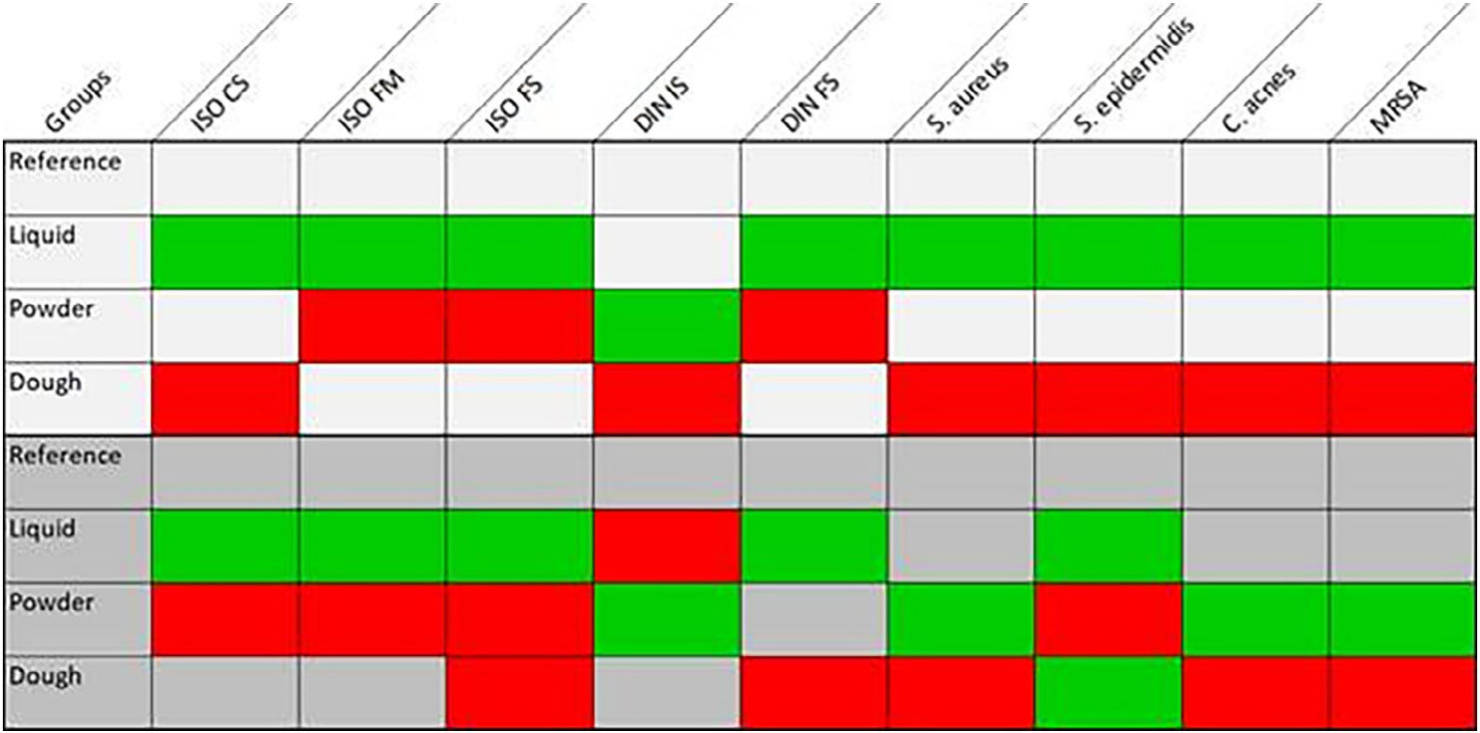
Summary of all test groups. ISO CS: ISO compression strength, green: moderate reduction (≤5 MPa), red: relevant reduction (>5 MPa); ISO BM: ISO bending modulus, green: moderate reduction (≤300 MPa), red: relevant reduction (>300 MPa); ISO BS: bending strength, green: moderate reduction (≤5 MPa), red: relevant reduction (>5 MPa); DIN I: DIN impact, green: moderate reduction (≤ 0. 5 kJ/m^2^), red: relevant reduction (>0.5 kJ/m^2^); DIN B: DIN bending, green: moderate reduction (≤5 MPa), red: relevant reduction (>5 MPa); efficacy after 24 h, green: moderate reduction (0–5 mm), red: relevant reduction (>5 mm).

In the present study, eight different cement mixtures were prepared and investigated. In what follows, number 1 indicates the references, 2 all cements after liquid clindamycin was added to the liquid cement compound, 3 all cements after liquid clindamycin was added to the cement powder, and 4 all cements after liquid clindamycin was added to the cement dough. (Author: Figs 1, 2, 3).

For product designation Basis PALACOS R (a): 1a R = reference, 2a R, 3a R, and 4a R. For product designation Basis PALACOS R + G (b): 1b R + G = reference, 2b R + G, 3b R + G and 4b R + G.

Each reference cement contained 1 g clindamycin powder; all test cements contained 1 mL clindamycin solution. All cements were produced by the PALAMIX System.

In the cases of reference groups 1a R and 1b R + G, 1 g clindamycin powder was mixed with 40 g PALACOS R or 40.8 g PALACOS R + G each and subsequently transferred to the PALAMIX System. Cements were produced according to the manufacturer's specifications. After curing, cements were poured into metal molds and a pressure of 3 bar was maintained for 30 min.

### Clindamycin Preparation

For all test groups, 1 g clindamycin powder was dissolved in 1 mL distilled water.

### Test Groups

In test group 2, 1 mL clindamycin solution was mixed with 20 mL monomer liquid with continuous stirring and then produced by the PALAMIX System with the appropriate cement powder.

In test group 3, the PALACOS R or PALACOS R + G polymer powder was mixed with 1 mL clindamycin solution by careful stirring in the PALAMIX System. Afterwards, the dough was produced with the monomer liquid.

In test group 4, the respective cements were produced by the PALAMIX System according to the manufacturer's specifications. After 15 sec the PALAMIX System was opened and 1 mL clindamycin solution was added and mixed with the mixing paddles for another 15 s.

### Mechanical Testing

Mechanical investigations were carried out according to ISO 5833: Implants for Surgery‐Acrylic Resin Cements (2002) and German Industry Standard/DIN 53435 (2007): Testing of Plastics. ISO 5833 specifies three different mechanical tests (CS, compression strength; FM, flexural model; FS, flexural strength), whereby special standardized molded bodies are used. The tests are carried out using special equipment with a number of repetitions (statistics) specified by the standard. The mean values are used.

Industry Standard/Deutsche Industrie Norm/DIN 53435 (2007) describes two different mechanical tests (FS, flexural strength; IS, impact strength), whereby special standardized molded bodies are used. The tests are carried out using special equipment with a number of repetitions (statistics) specified by the standard. The mean values are used.

Mechanical investigations were carried out at the Heraeus Medical laboratories in Wehrheim, Germany.

### Microbiology

For microbiological inhibitory tests, standardized cylindrical moldings (diameter: 25 mm, height: 10 mm) were produced and incubated in 10 mL buffer solution at room temperature for 24 h. All eluates were stored at 8°C in the fridge.

Agar plates were generated by spreading previously established suspensions of *Staphylococcus aureus*, *Staphylococcus epidermidis*, *Cutibacterium acnes*, and methicillin‐resistant *Staphylococcus aureus* (MRSA) with a 0.5 McFarland turbidity standard. A 6‐mm hole was punched centrally into the agar and 50 μL of one eluate was applied per plate. After inoculation, the plates were incubated for 24 h at 37°C.

Microbiological tests were carried out at the Medical University of Graz, Austria.

### Statistics

Data presentation, statistics, and graphs were carried out with GraphPad Prism 5.01 for Windows (GraphPad Software, San Diego California USA, www.graphpad.com).

## Results

### Investigation of International Standard Operations Mechanical Strength

A stronger reduction in mechanical strength could often be detected when liquid AB were added to the manufactured PMMA cements (test groups) compared to cases in which powdery AB were added (reference groups).

Among the various test groups, the ISO compression strength of groups 2a R and 2b R + G was like references 1a R and 1b R + G, respectively. Group 3a R differed only slightly from the reference, with a compression strength reduction of only 1.8%, while group 4a R expressed the greatest reduction (8.8%). Within group b, 3b R + G expressed the largest difference compared to reference 1b R + G, with a reduction of 6.3%. Test group 4b R + G showed a reduction of 1.7%.

**Figure 4:**
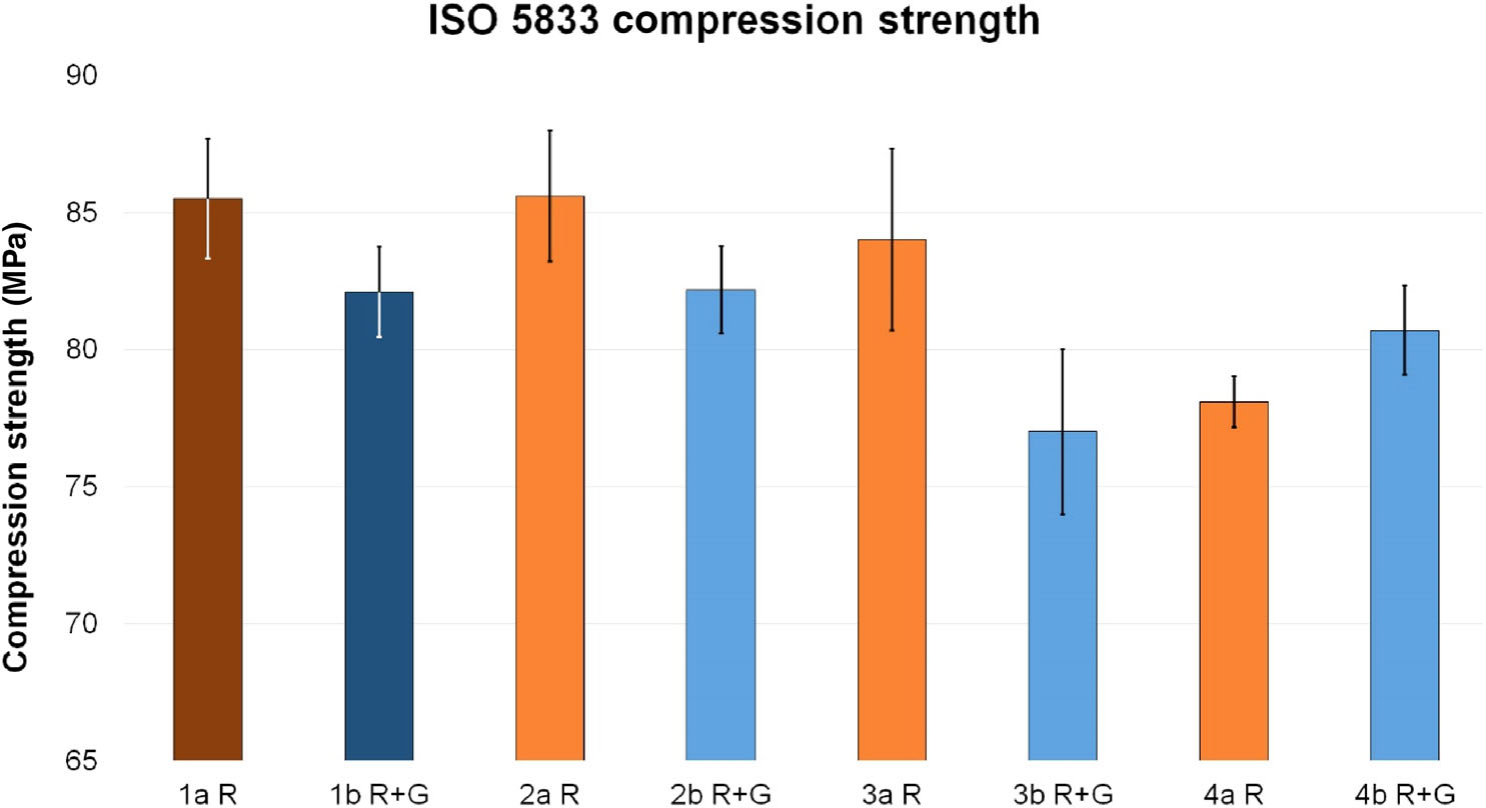
Average ISO compression strength (MPa) of reference groups 1a R (dark red, 85.51 ± 2.19) and 1b R + G (dark blue, 82.12 ± 1.64) and corresponding test groups, including standard deviations (group a light red, 2a R 85.6 ± 2.39, 3a R 84.01 ± 3.3, 4a R 78.09 ± 0.94; group b light blue, 2b R + G 82.18 ± 1.6, 3b R + G 77.01 ± 3.01, 4b R + G 80.71 ± 1.63).

The influence on the ISO compression strength of the investigated test groups was only minimal when liquid clindamycin was added to cement liquid. However, if liquid clindamycin was added to cement powder or dough, the strength values were comparatively lower (Fig. 4).

Regarding the ISO flexural modulus, all analyzed test groups differed from the reference groups. Group 2a R showed a reduction of 4.9%, group 3a R 12.9%, and group 4a R 11.5%. Within group b, group 2b R + G expressed the minimal difference compared to the reference, with a reduction of 1.4%, while test groups 3b R + G and 4b R + G showed a reduction of 12.5% each.

**Figure 5:**
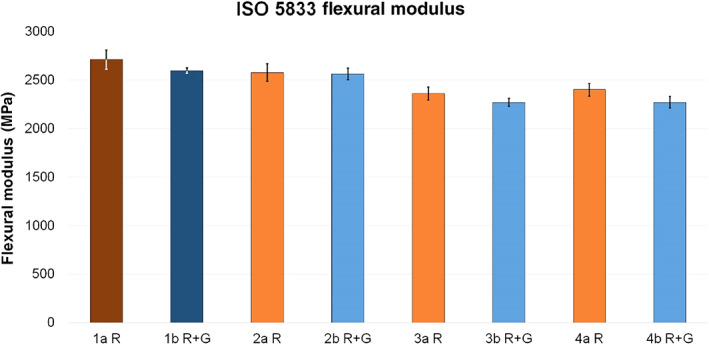
Average ISO flexural modulus (MPa) of reference groups 1a R (dark red, 2709 ± 100) and 1b R + G (dark blue, 2596 ± 28) and corresponding test groups, including standard deviations (group a light red, 2a R 2577 ± 89, 3a R 2358 ± 67, 4a R 2399 ± 64; group b light blue, 2b R + G 2560 ± 60, 3b R + G 2270 ± 40, 4b R + G 2270 ± 60).

With respect to the ISO flexural modulus, the strength values were again comparatively lower when liquid clindamycin was added to cement powder or dough instead of cement liquid (Fig. 5).

The determined strength values according to ISO flexural strength of test group 2a R and 2b R + G were the closest to the respective reference, with a reduction of 7.44% in the case of 2a R and 6.55% in the case of 2b R + G. Within group a, test group 4a R showed a strength reduction of 7.9% and test group 3a R of 10.9%. In group b, test group 3b R + G expressed the maximum reduction with 14.3%. Test groups 2b R + G and 4b R + G showed a reduction of 6.6% and 10.3%, respectively.

**Figure 6:**
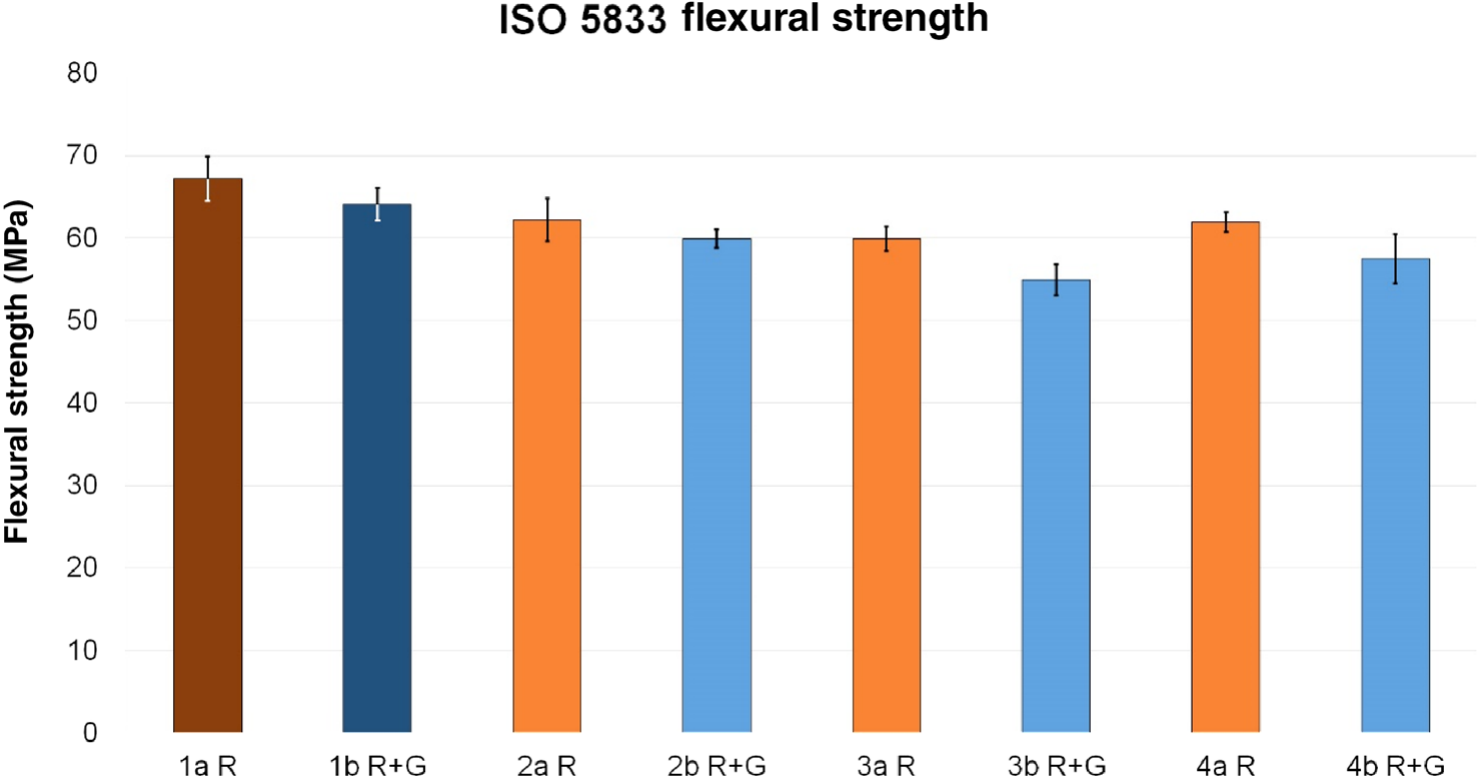
Average ISO flexural strength (MPa) of reference groups 1a R (dark red, 67.2 ± 2.7) and 1b R + G (dark blue, 64.1 ± 2) and corresponding test groups, including standard deviations (group a light red, 2a R 62.2 ± 2.6, 3a R 59.9 ± 1.5, 4a R 61.9 ± 1.2; group b light blue, 2b R + G 59.9 ± 1.1, 3b R + G 54.95 ± 1.93, 4b R + G 57.5 ± 3).

After addition of liquid clindamycin, the maximum reduction in flexural strength was detected when mixed directly with cement powder or cement dough **(**Fig.6 **)**.

### Investigation of Deutsche Industrie Norm Mechanical Strength

**Figure 7:**
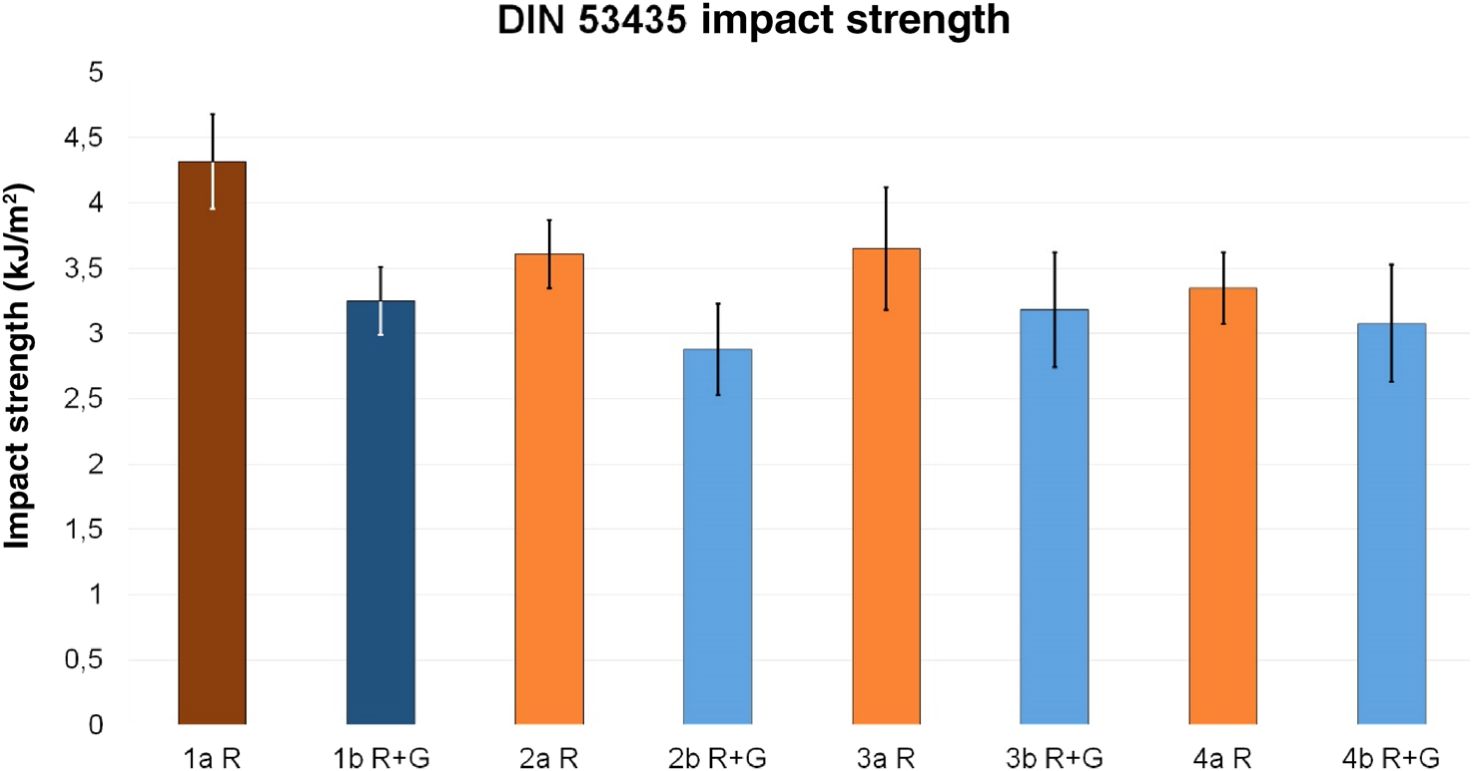
Average DIN impact strength (kJ/m^2^) of reference groups 1a R (dark red, 4.32 ± 0.36) and 1b R + G (dark blue, 3.25 ± 0.26) and corresponding test groups, including standard deviations (group a light red, 2a R 3.61 ± 0.26, 3a R 3.65 ± 0.47, 4a R 3.35 ± 0.27; group b light blue, 2b R + G 2.88 ± 0.35, 3b R + G 3.18 ± 0.44, 4b R + G 3.08 ± 0.45).

An analysis of the DIN impact strength revealed that the references showed the maximum impact strength compared to all test groups. The minimal difference to the corresponding reference was detected in test group 3a R, with a reduction of 15.5%. Test group 2a R showed a reduction of 16.4% and 4a R of 22.4%. Within group b, test group 3b R + G was the closest to its reference, with a reduction of only 2.2%, while test group 2b R + G showed a reduction of 11.4% and 4b R + G of 5.3% **(**Fig. 7 **).**

Regarding the DIN flexural strength, test groups 2a R (−6.9%) and 2b R + G (0.7%) were the closest to their corresponding references. Compared to reference 1a R, test groups 3a R and 4a R showed a reduction in flexural strength of 17.2% and 12.6%, respectively. Furthermore, test groups 3a R + G and 4a R + G showed a reduction in flexural strength of 17.2% and 12.6%, respectively, when compared to reference 1a R + G.

**Figure 8:**
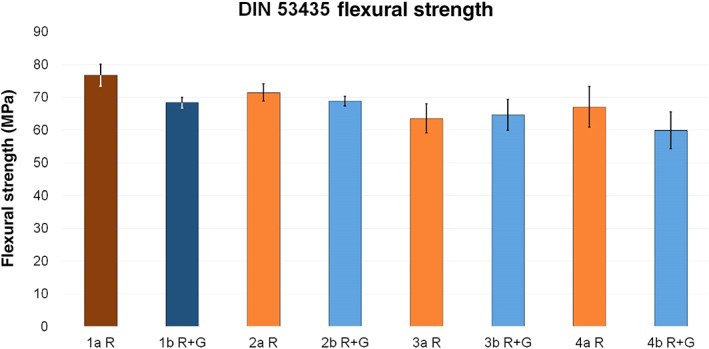
Average DIN flexural strength (MPa) of reference groups 1a R (dark red, 76.79 ± 3.37) and 1b R + G (dark blue, 68.38 ± 1.64) and corresponding test groups, including standard deviations (group a light red, 2a R 71.48 ± 2.59, 3a R 63.58 ± 4.46, 4a R 67.11 ± 6.22; group b light blue, 2b R + G 68.88 ± 1.48, 3b R + G 64.77 ± 4.69, 4b R + G 59.97 ± 5.6).

The minimal DIN flexural strength was detected when liquid clindamycin was added directly to cement powder or cement dough (Fig. 8).

### Microbiological Investigations

#### Efficacy against *Staphylococcus aureus*

In group a, the inhibition zone diameter was reduced by approximately 17.1% in test group 2a R compared to reference 1a R. Further on, the inhibition zone diameter continued to decrease by 27.3% in test group 3a R and by even 30.7% in 4a R.

**Figure 9:**
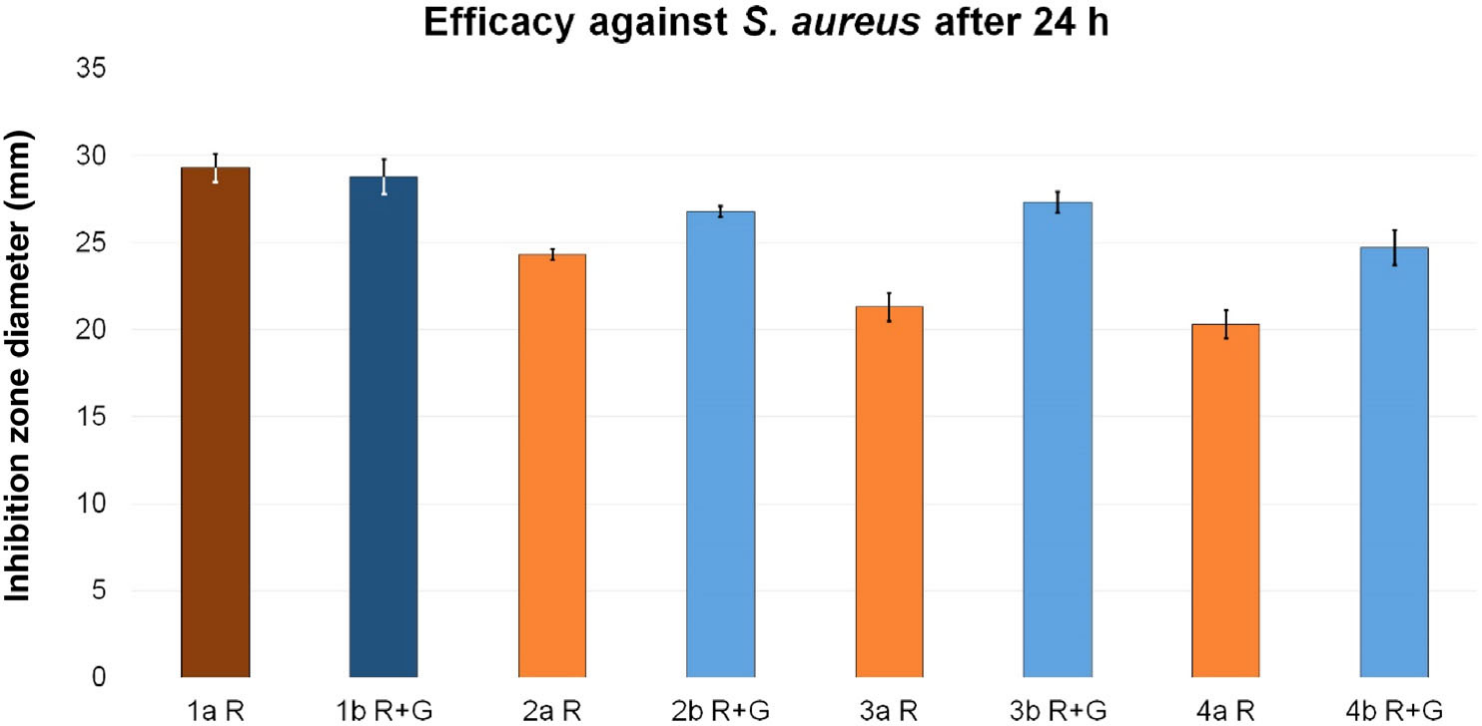
Inhibition zone test results for efficacy against *Staphylococcus aureus*. The inhibition zone diameters (mm) were measured after a 24 h incubation period for reference groups 1a R (dark red, 29.3 ± 0.8) and 1b R + G (dark blue, 28.8 ± 1) and corresponding test groups, including standard deviations (group a light red, 2a R 24.3 ± 0.3, 3a R 21.3 ± 0.8, 4a R 20.3 ± 0.8; group b light blue, 2b R + G 26.8 ± 0.3, 3b R + G 27.3 ± 0.6, 4b R + G 24.7 ± 1).

While a reduction of efficacy was detected in all test groups of group a (cement without gentamicin), the inhibition zone diameters of test groups b (cement with gentamicin) were similar to their reference 1b R + G. They were slightly reduced, by 5.2%, in test group 3b R + G and 6.9% in 2b R + G. The only exception was test group 4b R + G (admixture to cement dough), with a reduction of 14.2% (Fig. 9).

#### Efficacy against *Staphylococcus epidermidis*

In general, the inhibition zones detected in the test groups of group a were reduced compared to reference 1a R. Test group 2a R was the closest to the reference, with a reduction in the inhibition zone diameter of 19.2%. Test group 3a R showed a reduction of 38.7% and 4a R of 41.8%.

**Figure 10:**
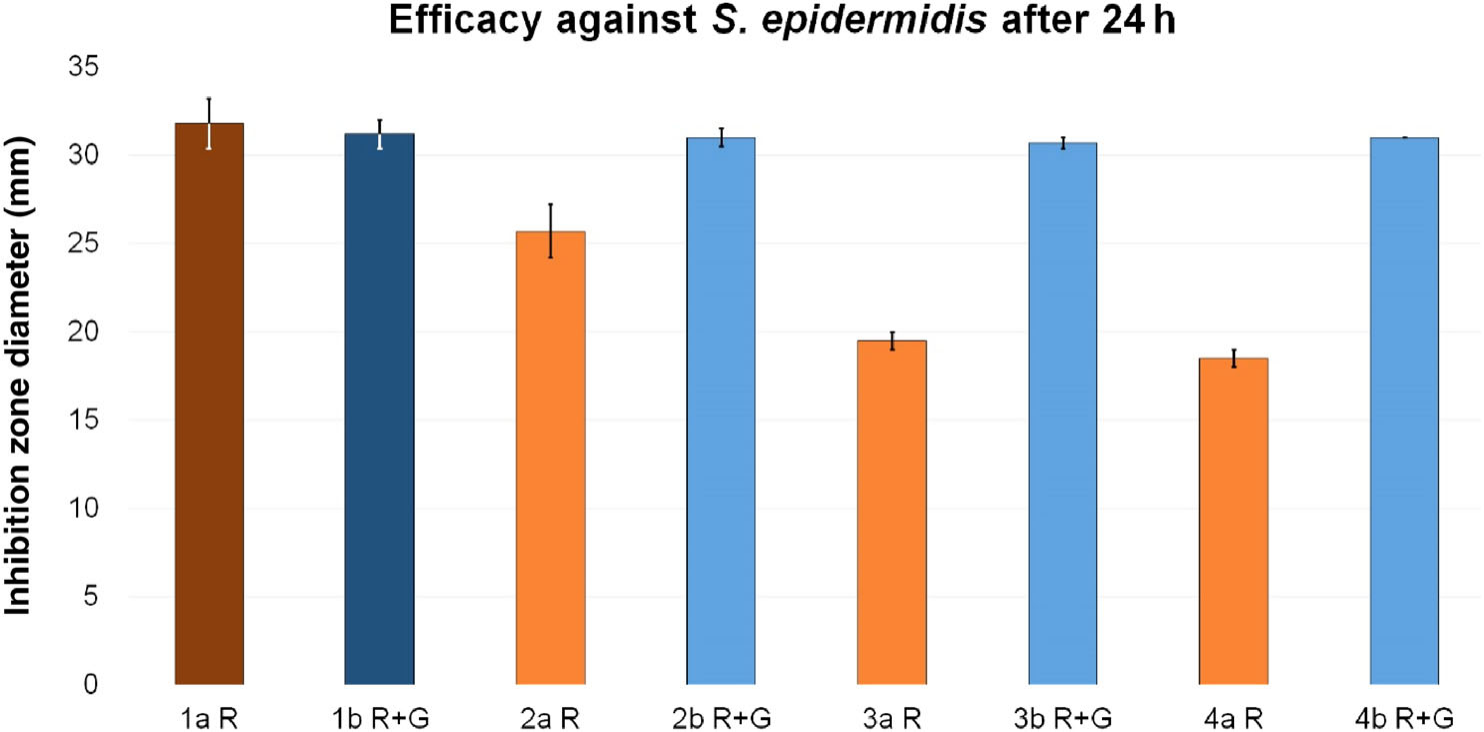
Inhibition zone test results for efficacy against *Staphylococcus epidermidis*. The inhibition zone diameters (mm) were measured after a 24 h incubation period for reference groups 1a R (dark red, 31.8 ± 1.4) and 1b R + G (dark blue, 31.2 ± 0.8) and corresponding test groups, including standard deviations (group a light red, 2a R 25.7 ± 1.5, 3a R 19.5 ± 0.5, 4a R 18.5 ± 0.5; group b light blue, 2b R + G 31 ± 0.5, 3b R + G 30.7 ± 0.3, 4b R + G 31 ± 0).

However, the inhibition zones detected in all test groups of group b were similar to reference 1b R + G (Fig. 10).

#### *Efficacy against* Cutibacterium acnes *after 24 h*

A comparison between group a (PALACOS R) and group b (PALACOS R + G) clearly demonstrated a reduced inhibition zone diameter when clindamycin was used without gentamicin.

**Figure 11:**
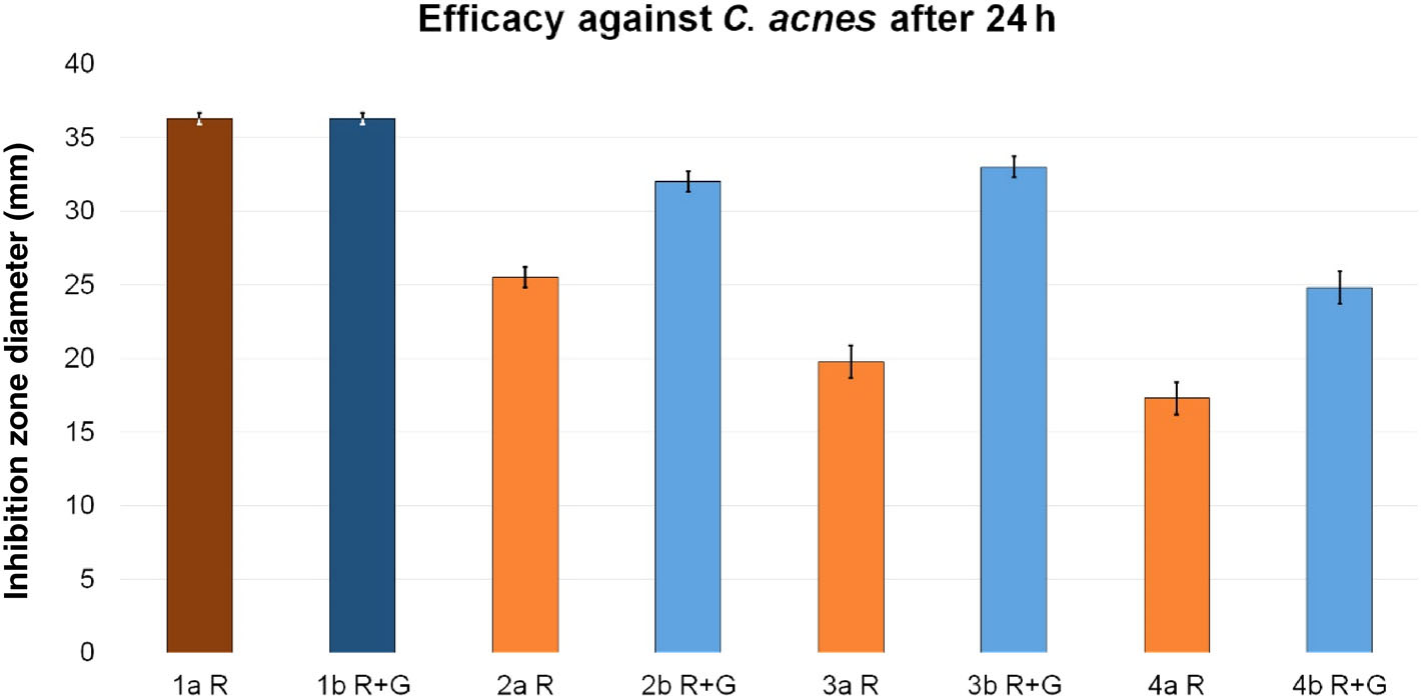
Inhibition zone test results for efficacy against *Cutibacterium acnes*. The inhibition zone diameters (mm) were measured after a 24 h incubation period for reference groups 1a R (dark red, 36.3 ± 0.4) and 1b R + G (dark blue, 36.3 ± 0.4) and corresponding test groups, including standard deviations (group a light red, 2a R 25.5 ± 0.7, 3a R 19.8 ± 1.1, 4a R 17.3 ± 1.1; group b light blue, 2b R + G 32 ± 0.7, 3b R + G 33 ± 0.7, 4b R + G 24.8 ± 1.1).

Within group a, test group 2a R showed a reduction of 29.8% in the inhibition zone diameter and was, thus, the closest to the reference. In test group 3a R, a reduction of 45.5% was detected. 4a R even expressed a further reduction of 52.4%. However, in group b, test group 3b R + G achieved the maximum inhibition zone diameter of all test groups, with a reduction of only 9.1% compared to the reference. The inhibition zone diameter was reduced by 11.8% in test group 2b R + G and by 31.7% in 4b R + G (Fig. 11).

#### *Efficacy against Gentamicin‐Resistant Methicillin‐Resistant* Staphylococcus aureus

When tested against gentamicin‐resistant MRSA, the inhibition zone diameters detected in group a (PALACOS R) were always reduced compared to those detected in group b (PALACOS R + G).

**Figure 12:**
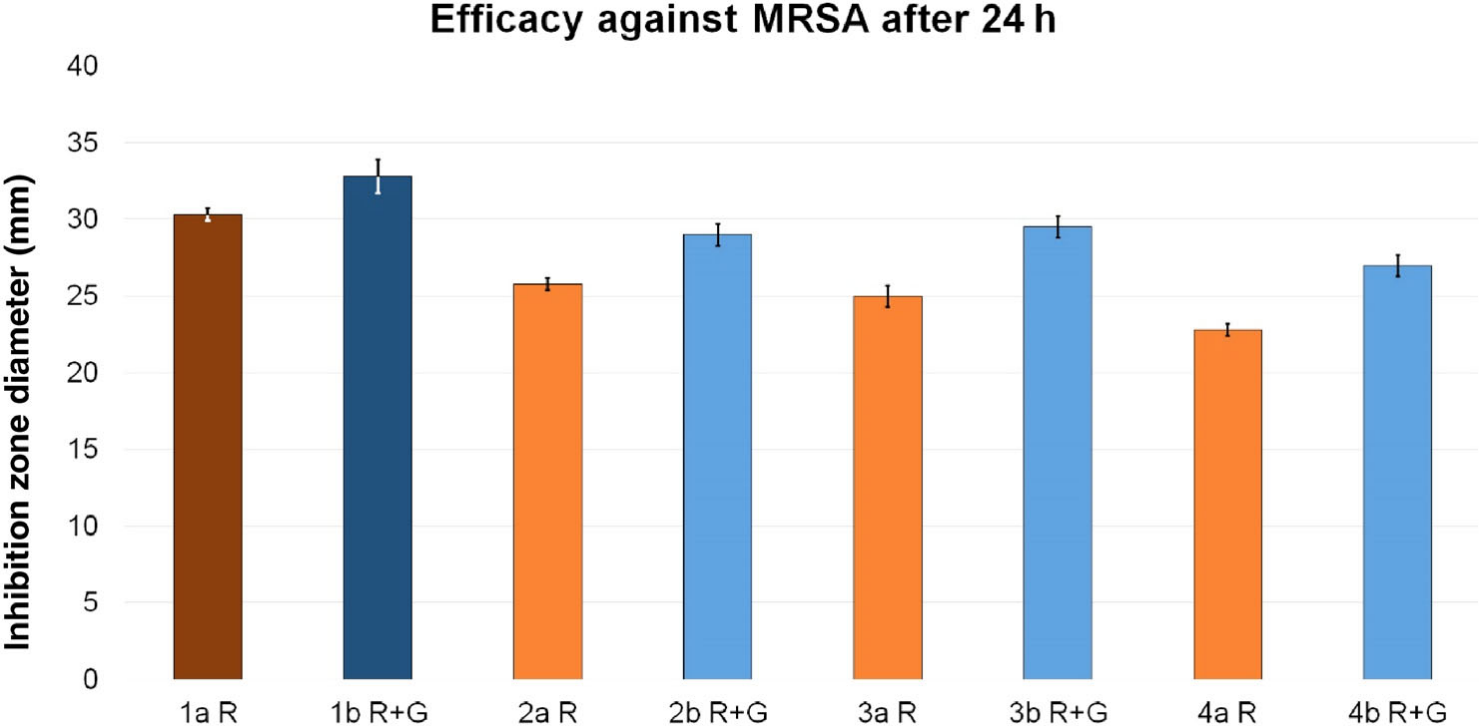
Inhibition zone test results for efficacy against methicillin‐resistant *Staphylococcus aureus* (MRSA). The inhibition zone diameters (mm) were measured after a 24 h incubation period for reference groups 1a R (dark red, 30.3 ± 0.4) and 1b R + G (dark blue, 32.8 ± 1.1) and corresponding test groups, including standard deviations (group a light red, 2a R 25.8 ± 0.4, 3a R 25 ± 0.7, 4a R 22.8 ± 0.4; group b light blue, 2b R + G 29 ± 0.7, 3b R + G 29.5 ± 0.7, 4b R + G 27 ± 0.7).

In group a, test group 2a R was the closest to reference 1a R, with a reduction of 14.9%. Test groups 3a R and 4a R showed a reduction of 17.5% and 24.8%, respectively. Meanwhile, in group b, the closest test group to reference 1b R + G was 3b R + G, with a reduction of 10.1% in inhibition zone diameter, followed by a reduction of 11.6% in 2b R + G and 17.7% in 4b R + G (Fig. 12).

## Discussion

In the present study, 1 mL liquid clindamycin was admixed manually into cement liquid, cement powder, and cement dough during the early phase of low viscosity.

Subsequently, the influence of the different ways to mix AB with cement on the mechanical stability of the implant was tested according to ISO 5833 or DIN 53435, and on microbiological efficacy by applying the inhibition zone test^17^.

The results clearly demonstrated that the addition of liquid anti‐infective agents to PMMA cement negatively affected mechanical and microbiological properties compared to the addition of powdery anti‐infective agents. This negative effect could already be detected at a low dose (1 mL of clindamycin) and is expected to increase significantly if higher doses are used. Furthermore, we expect that different types of anti‐infective agents will elicit a similar reduction of mechanical stability and microbiological efficacy if added as a liquid to PMMA cement.

Not all admixed (powdery) agents reduce the mechanical characteristics of PMMA^17^, ^32^, ^33^. Hydrophilic agents can potentially increase ISO compressive strengths. The higher the modulus, the less it is deformed^32^, ^33^, ^34^. If PMMA cement absorbs more liquid due to the admixture of anti‐infective agents, deformation under stress can increase. This, in turn, may result in a higher ISO compressive strength. The hydrophilic properties of the cement matrix are rarely affected by admixing liquid anti‐infective agents.

To assess the microbiological efficacy of admixed AB, reproducible standards are essential. However, if AB are admixed as liquids, a homogenous distribution within the cement remains questionable. In addition, the exact location of the admixed liquid AB within cured PMMA cement is unclear; if AB intercalate between polymer chains of the matrix, diffusion towards and release from the surface of the cement might be rather limited. Furthermore, the heat development during cement curing can lead to different evaporation rates of liquid AB. The temperature peaks are a serious issue, especially for the production of PMMA spacers outside the body, where the heat development cannot be buffered by prostheses, blood stream, and cement thickness. Liquid AB exposed to extreme heat during the spacer manufacturing process and temperature‐sensitive agents can easily be destroyed.

Low doses of liquid clindamycin were manually admixed to PALACOS cement in three different ways to examine at which doses the addition of liquid AB is feasible and how successful the procedure is. This exploration intends to establish a recommendation for surgeons, who may case‐dependently need to mix liquid AB, analogous to the preparation with powdery AB.

The admixture of the polar liquid clindamycin to the nonpolar methyl methacrylate (MMA) of the cement liquid will result in two practically immiscible phases (test group 2). How immiscible liquid plus cement powder distributes is strongly influenced by radically polymerizing, transforming liquid MMA into solid PMMA^17^, ^35^. The impact of the polar liquid clindamycin on the polymerization process is incidental. It will intercalate into the quickly forming polymer chains, affecting the mechanical stability of the cement matrix considerably.

During the wetting process of cement powder (polymer) with liquid clindamycin, the AB solution will be sucked up immediately by the dry polymer (test group 3). Thus, homogeneous wetting is not possible. The inhomogeneous distribution of AB is certainly one of the major reasons for the weakening of the cement matrix detected by the mechanical tests and weaker performance in the microbiological tests. A reproducible, homogeneous admixture of liquid anti‐infective agents during the early dough phase (15 s after dough preparation, test group 4) is impeded by the polymerization process, which has already started. A lot of polymer chains have already formed. Belated penetration of liquid AB is only selectively possible by using a paddle. Consequently pores will form in the cement matrix after curing, regarded as weak points^36^, ^37^, ^38^, ^39^, which are unfavorable for the agent release. They are not only localized on the surface but are also located deep within the cement matrix^40^.

We could demonstrate that admixing liquid anti‐infective agents to the cement liquid had the smallest negative effect on mechanics and efficacy.

The efficacy of AB containing PMMA cements is especially dependent on the diffusion ability of the anti‐infective agents used from the cement matrix^10^, ^11^, ^12^, ^41^. Antibiotic‐loaded cement is supposed to have a dual function: first, as a colonization barrier that protects the implant surface from germ invasion; and, second, as a measure to significantly reduce or eradicate the number of germs in surrounding tissues^7^, ^15^, ^42^.

Combining gentamicin and clindamycin meets the needs of local endoprosthetics because their germ spectra is complementary with a synergistic effect^12^, ^43^, ^44^, ^45^. The gentamicin/clindamycin combination successfully covers a spectrum of more than 90% of germs responsible for implant‐associated infections^14^, ^46^, ^47^ as verified by our microbial tests. Group b always displayed a greater effect compared with other test groups. Particularly in the case of *C. acnes* and MRSA, gentamicin alone is not or is rarely effective. The synergistic effect of the gentamicin/clindamycin combination was remarkable. The synergistic effect was best when clindamycin was added as a powder. Manual admixture of AB to PMMA cement is recommended for revision surgery upon infection and for therapy support. A concentration of 6 g powdery AB per 40 g PMMA cement has been shown to be sufficient^42^. Liquid AB have been discouraged because of their negative impact on mechanical properties^48^. For particular cases, the risk of mechanical disadvantages to preserve the joint and eradicating germs is acceptable. If prosthesis infection occurs necessitating spacer implantation, it prevents the patient putting pressure on the joint.

Admixing of up to 12 mL of liquid anti‐infective agents to the PMMA cement is possible. This admixture of liquid anti‐infective agents resulted in a 234% enhanced elution after 10 days^28^, although a mechanical reduction and a negative effect on the inhibition zones were detected during the previous period. This result is not explainable.

The present work confirms that an admixture of powdery AB is preferable to an admixture of liquid AB, which concurs with previous studies^41^, ^48^.

### Conclusion

If sterile powdery antibiotics are not suitable or available for the admixture to PMMA, liquid antibiotics may be used as an effective alternative. The best study results were achieved in test group 2 (liquid AB to liquid monomer), both mechanically and microbiologically.

If admixture of liquid AB is inevitable, the admixture of the liquid AB to the liquid monomer should be considered, followed by blending with the polymer powder. We discourage admixing liquid AB to PMMA powder or cement dough with low viscosity.

### Limitations

This study is limited because only one liquid antibiotic (clindamycin) was tested. A low concentration of clindamycin was admixed and investigated. Other antibiotics with various concentrations may have a different impact on the PMMA cement qualities.

## Disclosure

Prof. Dr Kühn reports personal fees from Heraeus Medical, outside the submitted work. This enabled mechanical investigations to be carried out at the Heraeus Medical in Wehrheim (Germany). None of the other authors has or may receive payments or benefits from Heraeus Medical related to this work.
